# Supplementary material for: Robotic Parenchymal-Sparing Pancreatectomy: A Systematic Review
Source: Cancers (Basel). 2023 Sep 1;15(17):4369. doi: 10.3390/cancers15174369 (PMC10486530; doi:10.3390/cancers15174369)
Supplement: Supplementary file 1 [file cancers-15-04369-s001.zip › cancers-2535588-supplementary.pdf]

File S1:Search Terms and Strategy

Protocol registered using INPLASTY

Article types to include:

- Clinical studies
- Comparative studies
- Clinical trials
- Observational studies

Article types to exclude:

- Guidelines
- Case series
- Case reports
- Lectures
- Review articles
- Systematic reviews +/- Meta-Analyses

Databases:

- PubMed
- Embase
- Google Scholar

Criteria:

- Study has to have >/= 5 patients enrolled
- Study must be published after the year 2001 (year of first reported pancreatic surgery)
- Study must be a retrospective comparison
- Enucleation to Enucleation
- Central to Central
- Synthesize the data from there

Exclude:

- Case reports
- Case series
- Reviews
- Other Systematic Reviews

Pancreas Search Terms:

((((((pancreas[MeSH Terms]) OR (pancreas[Title/Abstract])) OR (pancrea*[Title/Abstract])) OR (pancreatic[Title/Abstract])) OR (pancreat*[Title/Abstract])) OR (pancreatectomy[Title/Abstract])) OR (pancreatectomy[MeSH Terms])

Search #1: Pancreas

Surgery Search Terms:

Search #1: Robotic surgery

(((((minimally invasive surgical procedures[MeSH Terms]) OR (robot[Title/Abstract])) OR (robotic[Title/Abstract])) OR (robot*[Title/Abstract])) OR ("minimally invasive"[Title/Abstract])) OR ("minimally-invasive"[Title/Abstract])

Search #2: Enucleations & Centrals

((("parenchyma-sparing"[Title/Abstract]) OR ("parenchyma-preserving"[Title/Abstract])) OR ("parenchyma sparing"[Title/Abstract])) OR ("parenchyma preserving"[Title/Abstract])) OR (enucleation[Title/Abstract])) OR (enucleat*[Title/Abstract])) OR ("parenchyma spar*"[Title/Abstract])) OR ("parenchyma-spar*"[Title/Abstract])) OR ("parenchymal sparing"[Title/Abstract])) OR ("parenchymal-sparing"[Title/Abstract])) OR ("parenchymal-spar*"[Title/Abstract])) OR ("central pancreatectomy"[Title/Abstract])) OR ("central-pancreatectomy"[Title/Abstract])) OR ("central pancreat*"[Title/Abstract])) OR ("central-pancreat*"[Title/Abstract])) OR ("medial pancreatectomy"[Title/Abstract])) OR ("medial-pancreatectomy"[Title/Abstract])) OR ("medial pancreat*"[Title/Abstract])) OR ("medial pancreat*"[Title/Abstract])) OR ("middle pancreatectomy"[Title/Abstract])) OR ("middle-pancreatectomy"[Title/Abstract])) OR ("middle pancreat*"[Title/Abstract])) OR ("middle-pancreat*"[Title/Abstract])) OR ("intermediate pancreatectomy"[Title/Abstract])) OR ("intermediate-pancreatectomy"[Title/Abstract])) OR ("intermediate pancreat*"[Title/Abstract])) OR ("intermediate-pancreat*"[Title/Abstract])) OR ("meso-pancreatectomy"[Title/Abstract])) OR ("meso pancreatectomy"[Title/Abstract])) OR ("meso pancreat*"[Title/Abstract])) OR ("meso-pancreat*"[Title/Abstract])) OR ("parenchyma-sparing pancreatectomy"[Title/Abstract])) OR ("parenchyma-preserving pancreatectomy"[Title/Abstract])) OR ("parenchyma-sparing pancreat*"[Title/Abstract])) OR ("parenchyma-preserving panceat*"[Title/Abstract])) OR ("parenchyma-sparing pancreatic resection"[Title/Abstract])) OR ("parenchyma-preserving pancreatic resection"[Title/Abstract])) OR ("parenchyma sparing pancreatectomy"[Title/Abstract])) OR ("parenchyma preserving pancreatectomy"[Title/Abstract])) OR ("parenchyma preserving pancreat*"[Title/Abstract])) OR ("parenchyma sparing pancreat*"[Title/Abstract])) OR ("parenchyma sparing pancreatic resection"[Title/Abstract])) OR ("parenchyma preserving pancreatic resection"[Title/Abstract])

PubMed Filters:

- Year: 2001 – January 2023

- Articles: Full Text Available
- Article Type: Case Reports + Classical Articles + Clinical Trials + Comparative Studies + Multicenter Studies + Observational Studies
- Languages: English
- Preprints Excluded
